# Effect of Mn Doping on the Optical and Electrical Properties of Double Perovskite Sr_2_TiCoO_6_

**DOI:** 10.3390/ma15155123

**Published:** 2022-07-23

**Authors:** Nor Diyana Abdul Aziz, Alyea Sofea Kamarulzaman, Norazila Ibrahim, Zakiah Mohamed

**Affiliations:** 1 School of Chemistry, Faculty of Applied Science, Universiti Teknologi MARA, Shah Alam 40450, Selangor, Malaysia; 2 School of Physics, Faculty of Applied Science, Universiti Teknologi MARA, Shah Alam 40450, Selangor, Malaysia; alyeasofeakz@gmail.com (A.S.K.); noraz954@uitm.edu.my (N.I.)

**Keywords:** double perovskites, structural properties, optical properties, AC impedance, dielectric properties

## Abstract

A new series of Sr_2_TiCo_1−*x*_Mn*_x_*O_6_ (0.0 ≤ *x* ≤ 0.7) materials has been synthesized using the conventional solid-state method. In this research, X-ray diffraction (XRD) results showed that Mn was successfully doped at the Co site in a cubic structure with monoclinic *P*2_1_/*n* space group. The effect of Mn cation substitution on the structural, optical and electrical performance of Sr_2_TiCo_1−*x*_Mn*_x_*O_6_ double perovskite was investigated. The optical study revealed a nonlinearity pattern of the band gap that is referred to as the band gap bowing trend. Results from optical and Rietveld refinement supports that the band gap bowing trend is correlated with the charge distribution that produces unique effects on structural and size changes due to the Co-Mn compositions. The morphological scanning electron microscopy studies also showed that larger crystallite sizes were developed when dopant was added. Furthermore, increases in the conductivities support the lowering band gap of Mn-doped samples. Here, the intermixing of the atomic orbitals of Co-Mn provides an efficient interlink electrical pathway to improve conductivity and exhibits a high dielectric property at room temperature. These values are strong evidence that STCM material will be suitable for applications in the semiconductor industry.

## 1. Introduction

Complex oxides of perovskite structures have a general formula of *ABO*_3_, where the *A* site comprises alkaline or rare earth metal elements from group I or II, and the *B* site contains transition metal elements. This simple perovskite oxide exhibits excellent physical properties in applications as solid oxide fuel cells [1,2], batteries [3,4], parts of electronic devices [5], thermoelectrics [6,7], etc. More often, this group of perovskite serves as a reference material for studies due to its unique properties and potential applications in technologies interest. In the early 1950s, double perovskite oxide with a general formula of *AA*′*BB*′*O*_6_ (where *A* and *A*Ȃ = alkaline and alkaline earth or rare earth ions, respectively; *B* and *B*′ = transition metal ions) began to receive attention in the search for ferroelectric and piezoelectric materials [8]. Due to this, new series of double perovskite materials continue to receive a great deal of interest, for it offers flexibility and the unique ability to accommodate two ions of different types of radii and valences at the *B* site, thus creating a 3D checkerboard ordering of *BO*_6_ and *B′O*_6_ octahedrals.

Recently, prodigious attention has been given to Co-containing double perovskite oxide [8,9] and SrTiO_3_-based [10,11] oxide materials. This circumstance is due to the findings of Kobayasyi et al. and Muta et al. on admirable performance, particularly on colossal magnetoresistance and thermoelectric properties. Thus, tremendous efforts have been exerted to explore alternative double perovskites [1,6,12] by chemically altering the *B* and *B*′ = transition metal ions to find a high efficiency material for device applications. This phenomenon is attributed to the crystal structure, which can be defined either as cubic (*Fm*$\bar{3}$*m*), tetragonal (*I*_4_/*m*), monoclinic (*P*2_1_/*n*) etc., depending on the relative size of the B and B′ cations with respect to the A atoms. For instance, Manoun et al. [13] investigated the effect of temperature in the structure of Sr_2_CoWO_6_ double perovskite oxides when characterized by Raman spectroscopy. The first phase transition from monoclinic (*P*2_1_/*n*) to tetragonal (*I*_4_/*m*) crystal structure occurred at 80 °C, while the second transition to cubic (*Fm*$\bar{3}$*m*) occurred at 480 °C. Augsburger et al. reported that the structure of Sr_2_FeMoO_6_ is cubic above the ferrimagnetic Curie temperature, and below this temperature it undergoes a structural phase transition and becomes tetragonal (*I*_4_/m) [9]. Others also have reported a series of structural phase transitions evolving not only by isomorphous substitution of cations either at the *A* or *B* sites but also by valence compensation, with the cations having different chemical valency [14,15]. Reported research findings on doped materials have shown profound results regarding band gap energy, magnetic properties, electrical behavior, etc. [16,17,18], which may widen their applications. Thus, substitutional doping is a longstanding method that has been used to modify the characteristics of the materials without changing the crystal structure as the parent material. However, other characteristics, such as band gap and electrical and magnetic properties, will differ.

Scientists and academics are continuously looking for novel double perovskite materials that are scientifically intriguing to research. In this regard, this work has paid attention to a new series of Co-containing double perovskite Sr_2_TiCoO_6_ (STC) doped with Mn. In our literature search, few studies on substitution were carried out on the STC material, except the work by Tanmoy et al. which reported on Ca, La, and Mo substitution as promising dopant to be used for thermoelectric materials. A study on the Sr-based system of cobaltite is still lacking. Therefore, the focus in this study will be on Mn substitution in the Sr-based system of cobaltite. Here, the Goldschmidt tolerance factor [19] is used for designing new perovskite materials. The tolerance factor was calculated for Mn-doped Sr_2_TiCo*_x_*Mn_1−_*_x_*O_6_ double perovskites to predict structural distortion. Accordingly, investigation on the effect of Mn doping in the STC material was noteworthy. This situation is because of the substitution of Mn at Co site that will change the STC’s optical and electrical transport behavior. Therefore, the Sr_2_TiCoO_6_ and Sr_2_TiCo*_x_*Mn_1−_*_x_*O_6_ materials in this work will be synthesized by using the solid-state method. The aim in this study is to report on the structural, optical and dielectric properties of the Sr_2_TiCo*_x_*Mn_1−_*_x_*O_6_ materials. This study will portray the unique role of Mn in the system and its potential in technological applications.

## 2. Materials and Methods

### 2.1. Material Preparation

In this research, polycrystalline powder samples of a new series of Sr_2_TiCo*_x_*Mn_1−_*_x_*O_6_ (0.0 ≤ *x* ≤ 0.7) were synthesized by using the well-known solid-state reaction method. High-purity (≥99.99%) Sigma Aldrich SrCO_3_, TiO_2_, cobalt (II, III) oxide (Co_3_O_4_), and MnO_2_ powders were chosen as the starting materials to produce the Sr_2_TiCo*_x_*Mn_1−_*_x_*O_6_ compound. The mass of each starting chemical materials to be used were calculated and weighed according to the desired stoichiometric ratios. Then, all powders were mixed and ground by hand in an agate mortar with a pestle for approximately 1 h to achieve excellent homogeneity. After grinding, the mixed powder sample was then placed on an alumina crucible and calcinated in a furnace at 900 °C for 24 h. A heating rate of 15 °C min^−1^ and a cooling rate of 1 °C min^−1^ were set. Subsequently, the calcined powders were ground for another 1 h and left sintered in air at 1100 °C for 24 h, followed by slow cooling at 1 °C min^−1^. This step is important to maintain and ensure that the final product’s obtained stoichiometry is near the desired oxygen stoichiometry [20,21]. All final products of the Sr_2_TiCo*_x_*Mn_1−_*_x_*O_6_ series (where *x* = 0.0, 0.3, 0.5 and 0.7) were designated as STC, STCM3, STCM5, and STCM7, respectively, according to their Mn ratios. In the solid-state impedance spectroscopy (IS) studies, the samples were prepared with a pellet maker, ENERPAC Pellet Presser, at a pressure of 4-ton cm^−1^. The die used was from Specac (Britain) with a diameter of 1.0 cm that can withstand high pressure. The samples were sintered at 100 °C for 3 *h* to remove moisture and increase density.

### 2.2. Material Characterization

All the materials were first characterized by using the XRD patterns collected with an X-ray powder diffractometer. A PANalytical X’Pert Pro MPD diffractometer with a solid-state detector (accelerator) was used. The XRD measurements were performed using a Bragg-Brentano optical configuration in ambient conditions. The X-ray beam used was CuKα radiation. In this study, a high-quality dataset in which the highest peak above 10,000 counts as statistically required were obtained for a proper quantitative XRD analysis [22,23,24]. The structural studies of quantitative XRD patterns that measured over the 2θ range from 20° to 80° were analyzed by the general Structural Analysis System, and graphical user interface (i.e., EXPGUI) programs were used for Rietveld refinement. The morphology and crystallite size of the samples were examined using a field emission scanning electron microscope (FESEM), the JOEL JSM-7600F. The optical properties of the materials were studied with the Perkin Elmer Lambda 950 UV-vis-NIR spectrophotometer. The measurements were conducted in reflection mode using the diffused reflectance technique in ambient conditions. The Fourier-transform infrared (FTIR) spectra were measured on Varian Excalibur 3100 in the range from 2500–450 cm^−1^. All samples were run neat on a single refraction ZnSe crystal plate via an attenuated total reflectance sampling accessory. The solid-state impedance spectroscopy (IS) measurements were carried out using a WonATech WEIS510 Multichannel EIS system over the frequency range from 0.1 mHz to 1 MHz at room temperature. Samples were sandwiched between the stainless steel holder that was treated as a blocking electrode in the measurements. All data were analyzed with ZMAN software using a fitting algorithm with a suitable equivalent circuit. Later, the dielectric behaviors were determined from the impedance results.

## 3. Results and Discussion

### 3.1. Structural Analysis

**Figure 1:**
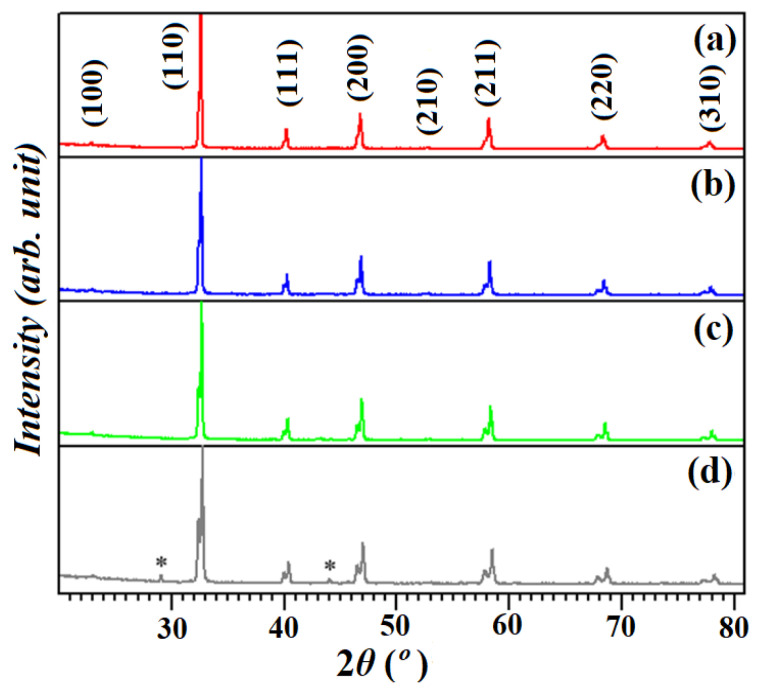
XRD results of (**a**) STC, (**b**) STCM3, (**c**) STCM5, and (**d**) STCM7 materials. The “*” mark indicates cobalt impurity phases presence in the sample.

The XRD patterns of all STCM samples demonstrating isostructural STC patterns are illustrated in Figure 1 and have been indexed according to their perovskite structures. The results in Figure 1a–c showed that the samples are pure and single-phase. In the case of STCM7 (the highest Mn concentration), two small peaks denoted by “*” indicate cobalt impurity phases at ~29° and ~44°, as shown in Figure 1d. Saxena et al. reported the same pattern, confirming the phase using X’pert Highscore with reference no. 98-0062716 [25].

**Figure 2:**
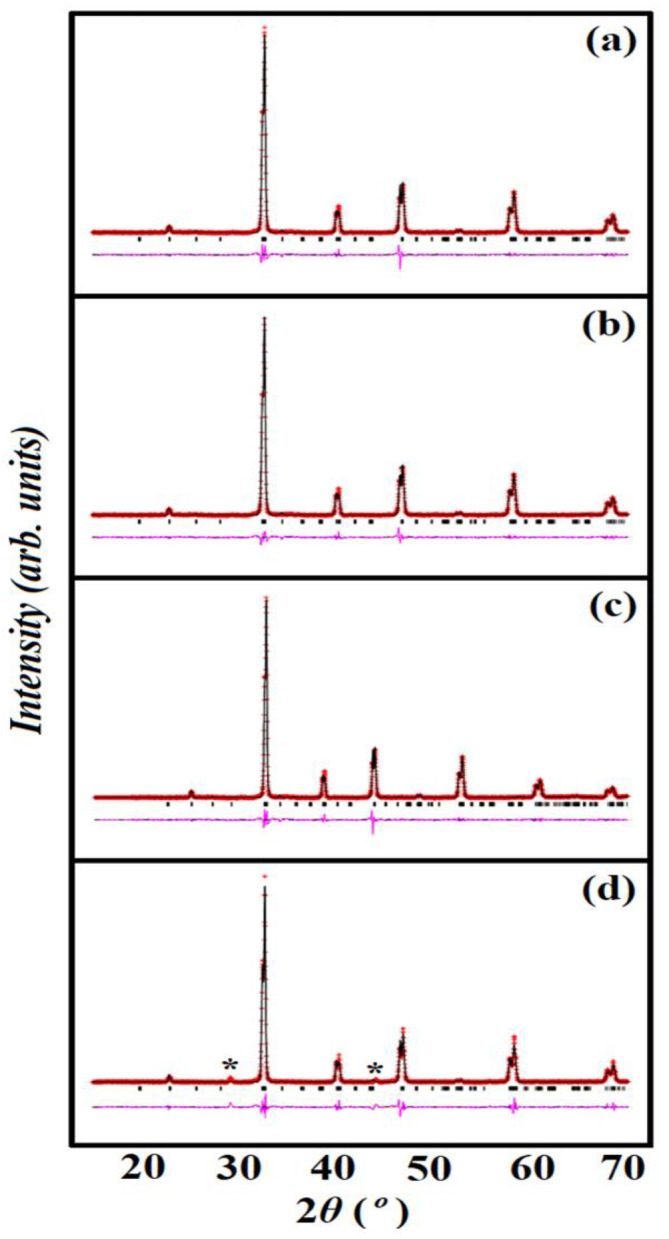
Rietveld refinement of XRD pattern of (**a**) STC, (**b**) STCM3, (**c**) STCM5 and (**d**) STCM7 samples. Black solid lines are observed data, the red solid line is the calculated pattern, and the pink solid line is the difference. Small black vertical tick marks above the difference plot denote the Bragg peak positions. The “*” mark indicates cobalt impurity phases presence in the sample.

The structural studies were carried out using the Rietveld method and GSAS software with the *P*2_1_/n space group, and results are displayed in Figure 2a,b. The final Rietveld refinement parameters are in the ranges of *R_wp_* = 0.0358 to 0.2587, *R_p_* = 0.0230 to 0.1504, and *λ*^2^ = 1.467 to 3.041, demonstrating that the structural parameters and agreement indices given in Table 1 are acceptable for powder materials. The lattice and refinement parameters *a* = 5.4861 Å, *b* = 5.5292 Å, *c* = 7.7474 Å, and cell volume (*V*) = 235.034 Å^3^ obtained for the STC sample are larger than those of Sr_2_TiCoO_6_ previously reported by Timothy et al. [26]. In particular, the refined unit cell volume (*V*) for each sample increased when Mn content increased in the pristine STC sample. The refined unit cell volumes for STC, STCM3, STCM5, and STCM7 are 235.034, 235.055, 235.342, and 235.569 Å^3^, respectively. This result suggests that Mn doping might have caused bonds in the A and B sites to elongate to accommodate the unaffected cubic tilting, increasing the lattice parameter and unit cell volume [25,26].

Information obtained from the XRD results was further used to determine the mean crystallite size (*D*) of each sample by using the Debye–Scherrer formula, as provided in Equation (1):

(1) $$D=\frac{k\lambda }{\beta cos\theta \prime }$$

where *k* is the shape factor, *λ* is the wavelength of the X-ray source, *β* is the full width at half maximum of the diffraction peak on a 2*θ* scale, and *θ* is the Bragg angle. The Scherrer formula was applied to the three main XRD lines, namely, (110), (200), and (211). The calculated mean crystallite sizes of STC, STCM3, STCM5, and STCM7 show an increasing trend from 75.8 nm, 86.0 nm, 87.9 nm, and 82.3 nm, respectively. This indicates that Mn-doped materials possess larger crystallite size and better crystallization than the undoped material. The tolerance factor (*τ*) of the compounds was calculated by using Equation (2):

(2) $$\tau =\frac{\frac{R_{a}+R_{a^{\prime }}}{2}+R_{o}\;}{\sqrt{2\;}\;\left(\frac{R_{b}+R_{b^{\prime }}}{2}+R_{o}\right)}$$

where *R_a_* and *R_a_*_′_ are the radii of the A site cations (Sr^2+^), *R_b_* and *R_b_*_𠄲_ are the radii of the B site cations (Ti^4+^/Co^4+^), and *R_o_* is the radius of the oxygen anion (O^2−^). The size of the ionic radius used was 1.44 Å (Sr^2+^) with CN:12 and 0.605 Å (Ti^4+^), 0.53 Å (Co^4+^), 0.53 Å (Mn^4+^), and 1.40 Å (O^2−^) with CN:6 [19]. The evaluated *τ* is presented in Table 1. The obtained *τ* value is 1.021 for all samples, indicating that the samples have ideal cubic structure and no structural distortion. This result is understandable because the choosing dopant of cations (Mn^4+^) has similar cationic radii to Co^4+^, and therefore, it is more captivating to investigate the effect of Mn doping in STC.

### 3.2. SEM Analysis

**Figure 3:**
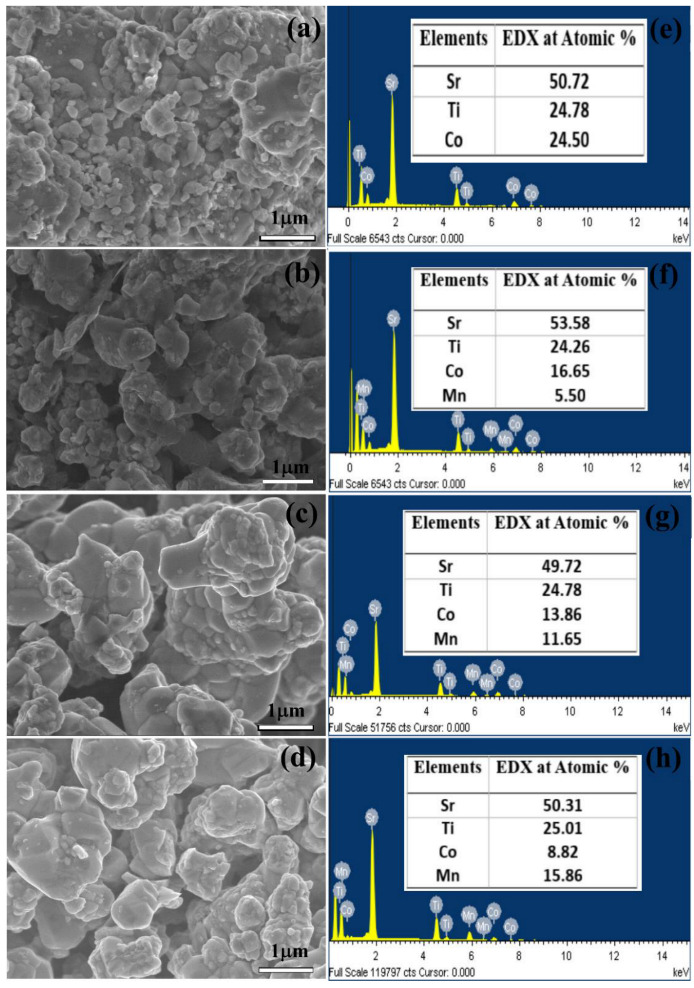
FESEM images of (**a**) STC, (**b**) STCM3, (**c**) STCM5, and (**d**) STCM7 materials at 20 *k* magnification; (**e**–**h**) are their corresponding EDX spectra, respectively.

**Figure 4:**
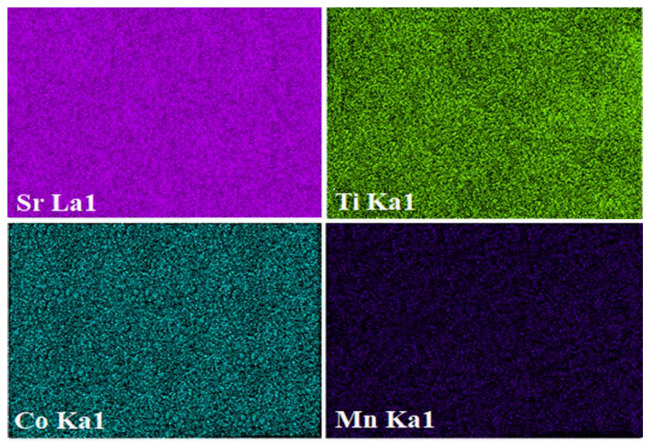
Energy dispersive X-ray spectroscopy (EDXS) of Sr_2_TiCo_0.7_Mn_0.3_O_6_ sample showing elemental mapping for constituents Sr, Ti, Co, and Mn.

The morphologies for all STCM samples (where 0.0 ≤ *x* ≤ 0.7) are shown in Figure 3a–d. All samples showed that the particles have irregular rounded crystal shapes. The presence of Mn^4+^ ions increased the small crystallite embedded in the larger chunks of materials. The doped samples displayed larger crystallite sizes compared to the undoped sample. This finding showed that the substitution of Mn in the STC material had caused in lessening of grain boundaries’ free energy. This led to the formation of larger grains. The average crystallite sizes for STC, STCM3, STCM5, and STCM7 are approximately 162.0, 165.2, 198.1, and 194.5 nm respectively. The crystallite size of all STCM samples was calculated using Image J software. The increment pattern of the crystallite size of Mn-doped samples with respect to its undoped sample supported the mean crystallite size calculated from the XRD results. The differences between these two methods were due to the mechanism of the measurement technique and sample size. The elemental analysis was carried out via EDX spectroscopy to determine the experimental stoichiometries of all samples. The results in Figure 3e–h show that the atomic percentages of all elements in the samples are in good agreement with the calculated stoichiometries. The EDX results have substantiated and supported the results from qualitative XRD. Accordingly, the XRD and EDX results showed that Mn has been doped in the STC material, which is direct proof that the substitutional doping was successful. Figure 4 shows that the color elemental mappings of the constituent elements, such as Sr, Ti, Co, and Mn, appearances on the selected area of the sample for the STCM3 material as a representative of all STCM samples. Although the distribution of these elements is difficult to precisely analyze, the mapping provides the notation that all the elements are well distributed throughout the sample matrix.

### 3.3. FTIR Analysis

**Figure 5:**
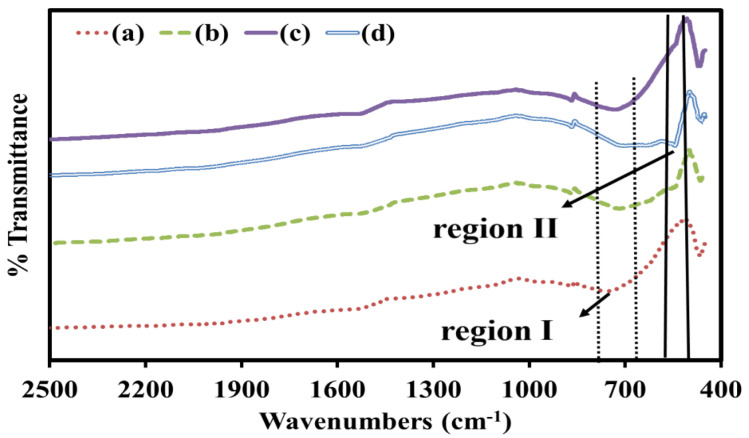
FTIR spectra of (**a**) STC, (**b**) STCM3, (**c**) STCM5, and (**d**) STCM7.

Figure 5 shows the compilation of FTIR spectra of all STCM samples that shown the appearance of new peak and a change in peak shape in the range from 450 cm^−1^ to 2500 cm^−1^. From the FTIR spectra, all the functional groups’ presence in the samples can be proven. The emergence of new peaks and peak shifting are expected, as they correspond to the specific vibration of functional groups and possible functional group interactions that took place due to addition of the dopant. The presence of the broad peaks between 739 cm^−1^ and 741 cm^−1^ (this range denoted as I) can be assigned to be the bridging vibration of the O-Co-O bond [27]. No significant changes in intensity are observed when more Mn is added, confirming that it is a O-Co-O bond formation and that it is present in all samples. A small observable peak occurs at 561 cm^−1^ (this range is denoted as II) when Mn dopant was introduced into the structure. This small peak corresponded to the characteristic stretching collision of O-Mn-O, demonstrating the presence of Mn in the sample [28]. This peak becomes clearer with increasing Mn content.

### 3.4. UV–Vis Analysis

**Figure 6:**
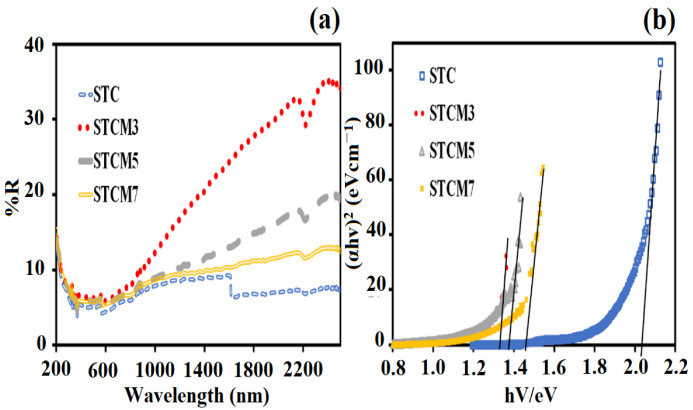
Results (**a**) UV–Vis NIR reflection spectroscopy and corresponding (**b**) Tauc plots and determination of band gaps of the STC and STCM materials.

In this study, in order to extract the optical band gaps, samples were measured by using the diffused reflectance technique, and all data were analyzed using the well-known Tauc relation [29] given below:

(3) $$\left(\alpha hv\right)=A{\left(hv-E_{g}\right)}^{x}$$

where *α* is the absorption coefficient of the material at a certain value of the wavelength *λ*, *h* is Planck’s constant, *A* is a proportionality constant, *υ* is the frequency of light, *E_g_* is the band gap energy, and *x* = ½ (for direct transition mode materials). The absorption coefficient is evaluated by using

(4) $$\alpha =k\;ln\left(\frac{R_{max}-R_{min}}{R-R_{min}}\right)$$

where *k* is a constant, *R_max_* is the maximum reflectance, and *R_min_* is the minimum reflectance. Consideration of Equations (3) and (4) gives

(5) $${\left(\alpha hv\right)}^{2}=A^{\prime }(hv-E_{g})$$

where *A*′ is a constant. According to Equation (5), a Tauc plot can be drawn of (*αhυ*)^2^ versus hυ. The point of the extrapolation of the linear part that meets the abscissa will provide the value of the band gap energy of the material. Figure 6a showed the UV–visible spectroscopic data, and Figure 6b showed the analyzed data obtained from the calculation. The obtained band gap value of the undoped STC is 2.03 eV, whereas band gaps of the Mn-doped samples are lower. These Mn-doped optical band gap values range from 1.33 eV to 1.45 eV (Table 2). This finding indicates that substitutional Mn doping has the ability to lower the band gap of the STC double perovskite compound. It is an established approach to add some impurity (doping) at a controlled rate to form new compounds with band-gap-narrowing properties [29,30].

**Figure 7:**
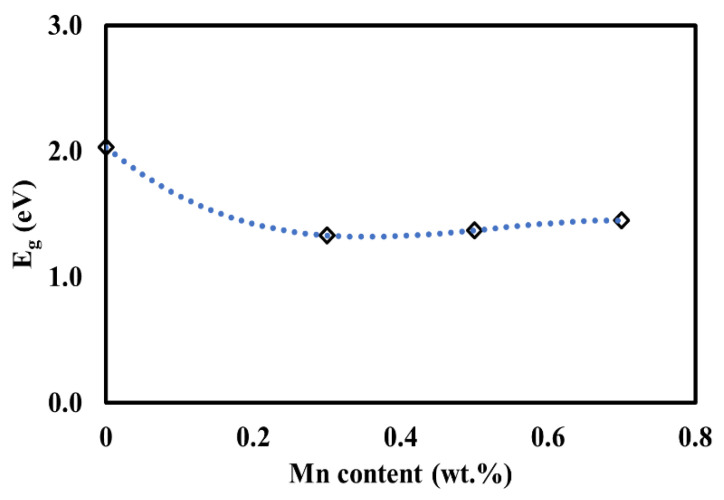
Graph showing the relationship between band gap energies and Mn concentration.

In this study, the decrease in the band gap of the Mn-doped samples with respect to the undoped samples is attributed to the presence of a mixture of Mn^2+^, Mn^3+^, and Mn^4+^ species in the Sr_2_TiCo*_x_*Mn_1−*x*_O_6_ crystal lattice. This phenomenon is attributed to Mn characteristics that can exist in many oxidation states. The presence of these Mn species (i.e., Mn^3+^ and Mn^4+^) is due to unpaired electrons in the *d* orbitals and thus resulted in band gap narrowing [31,32,33,34]. This is because the energy bands of doped samples are modified. The presence of the unpaired electrons from the 3 *d* transition metal of Mn ions results in crystal field splitting. Thus, Mn doping creates more energy levels in the band structure of Sr_2_TiCo*_x_*Mn_1−*x*_O_6_ materials, making them more conductive. This provides a promising development of new Mn−doped materials that will function as extrinsic semiconductors. However, there is a tuning of the band gap when Mn concentration is greater than (*x* ≥ 0.5) or when the material is starting to dope heavily. A similar finding of the band gap bowing phenomenon has been observed and reported by other researchers [35,36,37]. It is revealed that the band gap bowing is correlated with the extent of micro strain in its respective compound. This is consistent with the lattice parameter extension values obtained in Table 1. Here, Mn metal ion starting to reduce orbital overlap, moving the bands to deeper energies, and increases the band gap for heavily Mn−doped samples. This is due to the electron missing from five to seven electrons in their orbitals (depending on the oxidation state) has accounts for the large changes with respect to the Co 3 *d* transition metal. Therefore, the *d*−*d* transitions in the Mn−doped samples had caused the increased in the hybridization of the Co−Mn interaction and decreased the discreteness of the energy levels of the Sr_2_TiCo_1−*x*_Mn*_x_*O_6_ samples. A graph of the band gap versus Mn content in the STC structure is shown in Figure 7. The optical band gap obtained is decreased only when Mn content is up to 30%, followed by a linear increase with an increase in Mn concentration. Lower band gap values indicate that the Mn−doped samples should be more conductive than the STC sample.

### 3.5. AC Impedance Analysis

**Figure 8:**
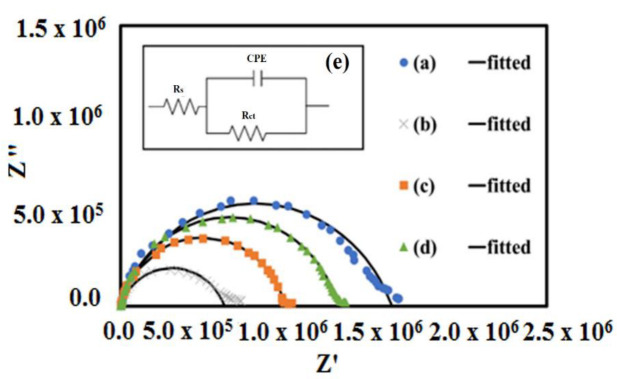
Nyquist plots of (**a**) STC, (**b**) STCM3, (**c**) STCM5, and (**d**) STCM7 and (**e**) circuit model used for curve fittings.

In order to investigate the effect of Mn on the electrical properties of STCM materials, impedance measurements were conducted and discussed below as a function of Mn content. AC impedance spectroscopy is widely used in order to separate out the true bulk conductivity from the conduction due to grain boundaries, electrode–sample interfacial impedance, and polarization effect within the materials. The measured complex impedance (*Z*) has real (*Z*′) and imaginary (*Z*″) components, which are interpreted as in Equation (6). The equation used is [38,39]

(6) $$Z=Z_{0}\;cos\phi +jZ_{0}\;sin\phi$$

where the real part *Z*′ = *Z*_0_ cosϕ and the imaginary part *Z*″ =j*Z*_0_ sinϕ. From the Nyquist plot (*Z*′ and *Z*″), impedance measurements were conducted and discussed. Figure 8a–d shows the Nyquist plots of all samples. All data are well fitted by using the ZMAN software. Here, the data interpretation of the Nyquist plots was performed by using the Randles equivalent circuit analog, as shown in Figure 8e, which depicts that all plots consist of one nearly perfect semicircle, indicating a single time constant or one Debye response. This semicircle is attributed to the existence of grain boundary aspects of the materials that affected the charge carrier movements. The scattered symbols used in Figure 8 are experimental data obtained, while the solid black lines are the fitted results from the equivalence circuit chosen by the software. It is observed from the equivalent circuit used that the parallel resistor (*R*) circuit along with constant phase element (*CPE*) represents the non-ideal capacitive behavior from Debye-type relaxation [40,41]. Ideally, a Debye-type response relaxation phenomenon will show that the perfect center of the semicircle overlaps with the real impedance axis. Thus, the Nyquist plots of all Sr_2_TiCo*_x_*Mn_1−*x*_O_6_ samples depict a non-Debye-type response. The bulk conductivity values of the samples were calculated using Equation (7):

(7) σ=t/A Rb

where *σ* is the conductivity; *t* is the pellet thickness; *A* is the contact area of pellet with the electrode; and *R_b_* is the bulk resistance, which is determined from the Nyquist plot.

Table 3 shows the tabulated data of the conductivity values of the STC and STCM materials at room temperature. The conductivity of the undoped STC sample is 8.69 × 10^−5^ S cm^−1^. It is observed that the conductivities of all Mn–doped samples were found to be higher than those of the pristine STC samples. This situation is due to Mn dopant provides additional sites or bridges, thus creating favorable high-conductivity pathways in the vicinity of grains for the migration of electrons. The maximum conductivity value achieved were at 2.63 × 10^−5^ S cm^−1^ where *x* = 0.3. The conductivity started to decrease when Mn content increased beyond *x* ≥ 0.5. The highest conductivity value achieved by the STCM3 sample was assisted by the poorly formed grain boundaries, which make it easy for the electrons to move across the bulk of the crystal. The SEM micrograph in Figure 3b shows that the grain boundaries of the STCM3 are not well-formed compared to the undoped and higher-Mn-doped samples. Meanwhile, the decrease in the conductivity values after optimum Mn concentration is due to a well-defined crystallite region or higher crystallinity. This phenomenon is due to the well-formed grain boundaries that normally impede the diffusion of electrons, thus decreasing conductivity [42]. The reported conductivity values in this study are relatively low compared to the results reported by Žužić et al. and Hernandez et al. [43,44]. This is due to selection of different synthesis processes and differences in calcination temperatures. However, the results obtained in this study are still comparable with other researchers’ reported values for *AA*′*BB*′*O*_6_double perovskite materials, like those reported by Debbebi et al. [45], Wiglusz et al. [46], and Digvijay et al. [47] on electrical conductivity values at room temperature: ~6.30 × 10^−3^ S cm^−1^, ~2.0 × 10^−4^ S cm^−1^ and ~2.51 × 10^−6^ S cm^−1^, respectively. From the impedance measurements, it is revealed that lightly Mn–doped (*x* = 0.3) Sr_2_TiCo*_x_*Mn_1−*x*_O_6_ sample showed higher electrical conductivity than any other Mn–doped samples with increasing concentration. This implies that Mn dopant plays a crucial role in electronic transport, as it creates more energy levels in the band structure of Sr_2_TiCo*_x_*Mn_1−*x*_O_6_ materials, making them more conductive. Thus, it provides the promising development of new Mn-doped materials that will function as perovskite semiconductors in technological applications.

**Figure 9:**
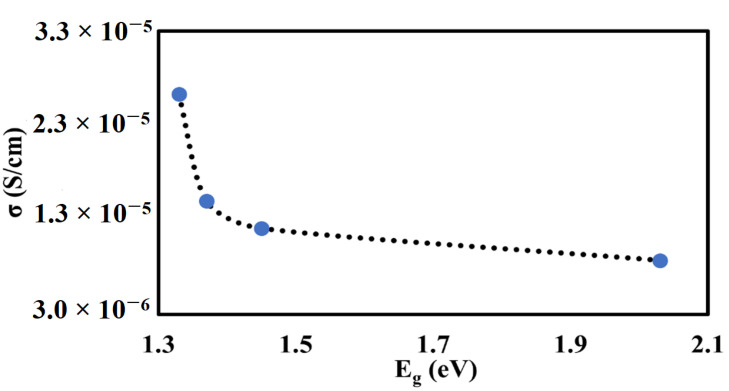
Graph showing the relationship of conductivity to band gap (E_g_).

Figure 9 is plotted to provide a better understanding of the correlation of conductivity with the band gap of the materials. The graph clearly shows that higher conductivities are more pronounced in the lower band gap region. This is because a low band gap means little energy is needed to jump into the conduction band. Thus, high conductivities are the manifestation of the lower band gap. 

### 3.6. Dielectric Analysis

**Figure 10:**
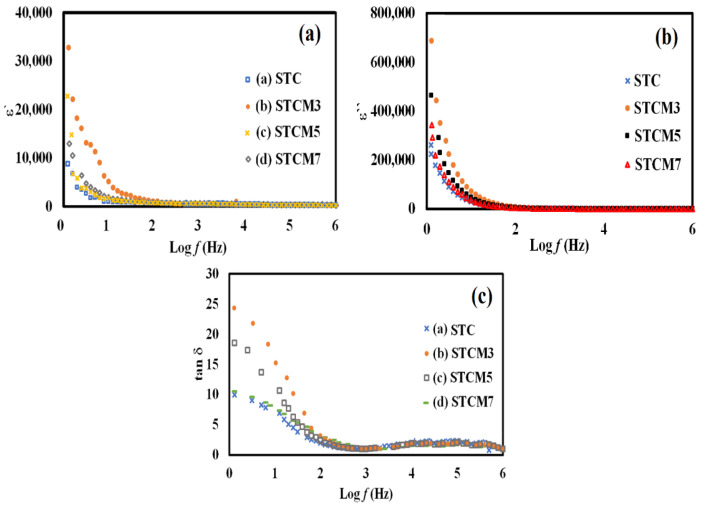
Graphs showing a (**a**) frequency dependence of *ε*′ (**b**) *ε*″ and (**c**) *tan δ* for STC and STCM materials at room temperature.

The dielectric relaxation behavior of the material provides important insights on the charge carrier transport phenomenon, this can be explained by modification of equation on the complex permittivity real parts and imaginary parts, as shown in Equations (8)–(11) [19]:

(8) $$\varepsilon^{*}\left(\omega \right)=\varepsilon^{\prime }\left(\omega \right)+j\varepsilon^{\prime \prime }(\omega )$$

(9) $$\varepsilon^{\prime }\left(\omega \right)=\frac{Z^{\prime \prime }}{\omega \varepsilon_{0}G|Z|²}$$

(10) $$\varepsilon^{\prime \prime }\left(\omega \right)=\frac{Z^{\prime }}{\omega \varepsilon_{0}G|Z|²}$$

(11) $$tan\delta =\frac{\varepsilon^{\prime \prime }}{\varepsilon^{\prime }}$$

where *ε*′ (*ω*) is the dielectric constant as a function of angular frequency (*ω*), which indicates the energy stored; *ε*″ (*ω*) is the energy loss during every cycle of the electric field; *Z´* and *Z*″ are the real and imaginary components of impedance, respectively; *ε*_0_ is the absolute permittivity (8.85 × 10^−12^ C^2^ N^−1^ m^−1^); and G is the area of electrode per thickness of pellet sample. Reliance of *ε*′ and *ε*″ on frequency can be correlated with various polarization effects, such as ionic, dipolar, electronic, and space charge, that appear at multiple levels of the material reaction due to short- and long-range movement of mobile chargers [32,33]. The variation of dielectric constant (*ε*′), dielectric loss (*ε*″), and tangent loss (*tan δ*) as functions of Mn concentration in terms of the frequencies at room temperature are shown in Figure 10a–c. Overall, from the graphs, it is observed that *ε*′ values are higher for Mn-doped samples than undoped samples. This may be related to the increase in free charge carriers in the samples. The dielectric constant values linearly decrease and merged in high frequencies. According to the Maxwell–Wagner interfacial polarization theory, the high dielectric constant obtained is due to the grains’ effect and interfaces [48]. At lower frequencies, the dielectric constant values are controlled by the grain boundary phases. The conductivity of the grain boundary is more active at lower frequencies, as the grain effect becomes dominant at high frequencies. This phenomenon is also attributed to the space charge polarization that primarily consists of the accumulated heavy electrical dipoles at grain/grain boundary interfaces when electrical field alternation occurs [49,50]. The dielectric constant value remains unchanged because the electron/hole exchange will not occur at high frequency [48,51,52]. In this high-frequency region, the real part of permittivity is tiny or negligible due to lagging with the applied field. The dielectric constant (*ε_r_*) obtained in 1 < log *f* < 6 is in the following sequence: *ε_r_* (STC) < *ε_r_* (STCM7) < *ε_r_* (STCM5) < *ε_r_* (STCM3). This finding is in accordance with the conductivity variation in the STCM system, as presented in Figure 8. A steep drop in *ε*′ in the low frequency range is supported by high losses in *tan δ* at the same frequency range as in Figure 10c. *Tan δ* illustrates the energy loss in the compounds in the midst of electric field alternation. The tangent loss value is large in the lower frequency range and slowly merges with increasing frequency. Furthermore, the tangent loss increases with increasing conductivity values at lower frequencies. This is due to the presence of activated chargers and the accumulation of charge carriers from the dopant. The tangent loss linearly decreases with an increase in frequency. According to Koop’s theory, the loss is reduced to saturation at higher frequencies when the resistivity is low due to more dominant conducting grains; hence, minimum energy is required to exchange electron between two ions, such as Co and Mn [53]. The results of dielectric constant (*ε*′), dielectric loss (*ε*″), and tangent loss (*tan δ*) are tabulated in Table 4. According to the results, the STCM3 sample has shown the highest dielectric constant (33157.9 ± 0.1) and dielectric loss (463283.8 ± 0.1) values and low tangent loss (24.2 ± 0.1) at 0.1 Hz. These results suggest that this substitutional doping of Mn into the STC material can significantly alter the dielectric properties of cobaltite-based double perovskites.

## 4. Conclusions

A new series of Mn–doped Sr_2_TiCo_1−*x*_Mn*_x_*O_6_ (0.0 ≤ *x* ≤ 0.7) double perovskite materials was successfully synthesized by the solid-state reaction method and crystallized into a cubic structure with a *P*2_1_*/n* space group. The IR spectra exhibited the characteristic bands of the O-Co-O bonds, confirming the fingerprint structure of this double perovskite. The SEM images revealed that the grain size increased when dopant was added up to *x* ≥ 0.5 and decreased when *x* = 0.7. Moreover, the band gap narrowing occurred in all Mn-doped samples with respect to their undoped materials. However, the trend of band gap widening was observed in Mn-doped samples. This phenomenon indicated that many bodily interactions occurred in the visible range, resulting in narrowed and widened samples. The AC impedance results confirmed that the non-Debye-type of relaxation phenomena occurred in all samples. Here, the increase in conductivity values is accompanied by a decrease in band gap. The electrical properties that are determined by impedance characterization also revealed that STCM3 exhibited the highest *ε*′ (33.2 × 10^3^) at room temperature. This finding showed that the characteristics of Sr_2_TiCo_1−*x*_Mn*_x_*O_6_ can be altered by a substitutional doping method and suggests that these materials could be promising candidates to be used in electronic devices because of their semiconductor optical band gap and excellent electrical behavior.

## Figures and Tables

**Table 1:** Crystallographic parameters of the STC and STCM samples (where 0.0 < *x* < 0.7) from the Rietveld refinements of the XRD data sets.

Doping Content (*x*)	STC (*x* = 0.0)	STCM3 (*x* = 0.3)	STCM5 (*x* = 0.5)	STCM7 (*x* = 0.7)
Lattice parameter				
Space group	*P*2_1_*/n*	*P*2_1_*/n*	*P*2_1_*/n*	*P*2_1_*/n*
Symmetry	monoclinic	monoclinic	monoclinic	monoclinic
*a (Å)*	5.4861	5.4873	5.5001	5.5039
*b (Å)*	5.5292	5.5315	5.5253	5.5200
*c (Å)*	7.7474	7.7441	7.7434	7.7539
*a = β = γ*	90	90	90	90
Unit cell volume (*Å*)^3^				
Unit cell volume	235.034	235.055	235.342	235.569
Goodness of fit				
*λ* ^2^	1.467	2.012	1.451	3.041
*R_p_*	0.0230	0.0345	0.0471	0.1504
*R_WP_*	0.0358	0.0506	0.0858	0.2587

**Table 2:** The band energy of the STC and STCM materials.

Samples	Band Gap Values (eV)
STC	2.03
STCM3	1.33
STCM5	1.37
STCM7	1.45

**Table 3:** The conductivity values of the STC and STCM materials.

Samples	Average *R_b_* (Ω)	Conductivity, (*S*/cm) at 25 °C
STC	1.67 × 10^6^	8.69 × 10^−6^
STCM3	5.59 × 10^5^	2.63 × 10^−5^
STCM5	9.69 × 10^5^	1.50 × 10^−5^
STCM7	1.18 × 10^6^	1.21 × 10^−5^

**Table 4:** The dielectric constant, (*ε*′); dielectric loss, (*ε*″); and loss tangent, *tan δ* values at 0.1 Hz of STC and STCM materials.

Samples ID	Dielectric Constant, *ε*′ at 0.1 Hz	Dielectric Loss, *ε*″ at 0.1 Hz	Loss Tangent, *tan δ* at 0.1 Hz
STC	88.0 × 10^2^	46.3 × 10^4^	9.0
STCM3	33.2 × 10^3^	68.9 × 10^4^	24.2
STCM5	23.1 × 10^3^	34.4 × 10^4^	17.3
STCM7	13.1 × 10^3^	26.1 × 10^4^	12.1

## Data Availability

Not applicable.
